# F94. A PREVALENCE PILOT STUDY FOR OBSTRUCTIVE SLEEP APNEA SYNDROME IN CLOZAPINE USERS

**DOI:** 10.1093/schbul/sby017.625

**Published:** 2018-04-01

**Authors:** M F Knevel, L B Luteijn, Jan Bogers, Jean-Paul Selten

**Affiliations:** 1 GGZ Rivierduinen; 2 GGZ Midden Holland; 3 Rivierduinen Centre for Mental Health, Dutch Clozapine Collaboration Group; 4 Maastricht University, Rivierduinen Mental Health Care Institute

## Abstract

**Background:**

Obstructive Sleep Apnea Syndrome (OSAS) is a frequent and common disorder. Estimated 50.000 persons in the Netherlands suffer from this disorder. Clozapine is known for its efficacy in treatment resistant schizophrenia. Frequent side effects of clozapine are weight gain, fatigue, sleepiness and metabolic syndrome. Similar symptoms occur in the course a OSAS. The Dutch pharmacovigilance centre LAREB (LAREB 2012) proposed an association between OSAS and clozapine usage, independent of confounding factors as obesity, smoking and glucose intolerance. Although clozapine is much used in the treatment of schizophrenia, OSAS prevalence studies in the clozapine treatment group are scarce. Research is needed to elucidate the relationship between clozapine use and OSAS. Identifying OSAS and treatment with continuous positive airway pressure (CPAP) could possibly (Galletly te al, 2016), through reduction in cardiovascular risk factors, have a favorable effect on mortality and possibly have a positive effect on daytime sleepiness, fatigue and daytime functioning.

Primary goal of this study is discovering the prevalence of OSAS in clozapine using schizophrenic spectrum disorder patients. The secondary goal is discovering how willing schizophrenic spectrum disorder patient are in undergoing a polysomnography.

**Hypothesis:**

Many patients with schizophrenia spectrum disorder have multiple OSAS risk factors: obesity, presence of metabolic syndrome, frequent usage of benzodiazepines, male sex, older age. OSAS prevalence is estimated to be much higher than in the general population because of these risk factors. Atypical antipsychotics are an independent risk factor for the development of OSAS. Polysomnographically diagnosed OSAS will even be higher in the clozapine treatment group estimated to be present in 30% percent of the patients.

**Methods:**

Research design: prospective observational and cross-sectional study in a group of stable adult patients with DSM IV schizophrenia spectrum disorder treated with clozapine in an outpatient community mental health service. Estimated study group consists of 30–50 patients. Exclusion criteria: unwillingness to undergo a polysomnography, inability to give informed consent, insufficient understanding of the Dutch language, severe cardiac failure, a history a cerebrovascular accidents and alcohol abuse.

**Method:**

screening on the presence of OSAS symptoms and risk factors associated with OSAS through: Epworth Sleepiness Scale for daytime sleepiness (Johns, 1991), STOP-BANG Questionaire ((SBQ: Chung 2012) when there is a high risk for OSAS followed by an ambulatory polysomnography including heart rate/ECG, respiratory measures with nasal flow canule and thermistor flow inductive respiratory movements, oximetry, and snoaring noises through sensory measurements (AASAM, 2009). OSAS is considered to be present in the presence of daytime sleepiness and if the Apnea Hypopnea Index (AHI) is larger than 5.0 obstructive or mixed type respiratory events per hour (AASM, 2009: Berry et. al, 2015: NVALT & CBO, 2009).

Statistical analysis: polysomnography: descriptive and univariate analysis. Presence of OSAS will be dichomotized (1 =OSAS present; 0 = OSAS absent) Summation of the amount of positive results will be presented as percentage of the total study population. Chi- squared test for considering of the height of the results on the ESS test and the STOP-Bang test and the prevalence of OSAS. Statistical significance: p < 0.05.

Prevalence: percentage of patients in the clozapine treatment group with OSAS.

**Results:**

No results at the moment of poster submission. In April 2018 results will be presented.

**Discussion:**

Will be presented in April 2018.

